# A novel isolation method for cancer prognostic factors via the p53 pathway by a combination of *in vitro* and *in silico* analyses

**DOI:** 10.18632/oncoscience.411

**Published:** 2018-04-29

**Authors:** Yohey Kamijo, Kohichi Kawahara, Takuma Yoshinaga, Hiroyuki Kurata, Kazunari Arima, Tatsuhiko Furukawa

**Affiliations:** ^1^ Department of Molecular Oncology, Graduate School Medical and Dental Sciences, Kagoshima University, Kagoshima 890- 8544, Japan; ^2^ Department of Chemistry and Bioscience, Faculty of Science, Graduate School of Science and Engineering, Kagoshima University, Kagoshima 890-0065, Japan; ^3^ Division of Clinical Application, Nanpuh Hospital, Kagoshima 892-8512, Japan; ^4^ Department of Bioscience and Bioinformatics, Kyushu Institute of Technology, Fukuoka 820-8502, Japan

**Keywords:** cancer, p53, tumor-suppressor pathway, short-hairpin RNA, genome analysis

## Abstract

Identifying new therapeutic target genes affecting the survival of patients with cancer is crucial for the development of new cancer therapies. Here, we developed a novel technology combining *in vitro* short hairpin RNA (shRNA) library screening and *in silico* analysis of the tumor transcriptome to identify prognostic factors via the p53 tumor-suppressor pathway. For initial screening, we screened 5,000 genes through selection of shRNAs in p53 wild-type tumor cells that altered sensitivity to the p53 activator actinomycin D (ActD) to identify p53 regulatory genes; shRNAs targeting 322 genes were obtained. Among these 322 genes, seven were prognostic factor candidates whose high expression increased ActD sensitivity while prolonging the survival period in patients with the p53 wild-type genotype. Conversely, we identified 33 genes as prognostic factor candidates among ActD-resistant genes related to a shortened survival period only in p53 wild-type tumors. These 40 genes had biological functions such as apoptosis, drug response, cell cycle checkpoint, and cell proliferation. The 40 genes selected by this method contained many known genes related to the p53 pathway and prognosis in patients with cancer. In summary, we developed an efficient screening method to identify p53-dependent prognostic factors with *in vitro* experimental data and database analysis.

## INTRODUCTION

*TP53* is one of the most commonly inactivated genes in human cancer; the gene product, p53, acts as a tumor suppressor. P53 promotes the transcription of a variety of genes, including genes encoding BCL2 associated X, apoptosis regulator *(BAX)* [1] and p53 upregulated modulator of apoptosis *(PUMA)* [2], which induce apoptosis, P21 [3] and *14-3-3σ* [4], which arrest the cell cycle; and growth arrest and DNA damage-inducible 45 *(GADD45)* [5,6], which repairs DNA damage. This variety of target genes confers p53 with tumor- suppressive functions. Under normal conditions, p53 protein levels are kept low by degradation via mouse double minute 2 homolog (MDM2), an E3 ubiquitin ligase for p53 [7,8]. Stresses such as radiation, DNA damage agents, or RNA synthesis inhibitors (e.g., actinomycin D [ActD]) [9-11], as well as oncogenic stress induced by abnormal activation of oncogenes, including Ras [12], and nutrient starvation [13] inhibit MDM2 function, resulting in accumulation and activation of p53 and p53-dependent cellular responses [14]. P53 is mutated and shows loss of transcription factor activity in about half of all cancers [15]. Moreover, p53 mutational status also greatly affects the survival of patients with cancer [16,17]. For example, the expression of genes such as *PICT1*, which functionally regulates p53 activation, affects survival in patients with colorectal, esophageal, gastric, and lung cancers [18- 21]. Moreover, novel p53 regulatory mechanisms, such as modulation of the nucleolar stress response, have also recently been revealed [22]. Thus, from the continuing evolution of our knowledge of p53, it is clear that the molecular mechanisms regulating complex p53 signaling pathways have not yet been fully clarified.

In recent years, high-throughput genomic analysis methods, such as next-generation sequencing (NGS) and genomic-scale microarrays, have been developed. Consequently, large amounts of information regarding the cancer genome and transcriptome have been obtained. These datasets are available from The Cancer Genome Atlas, Gene Expression Omnibus (GEO), and other databases and have greatly contributed to recent developments in tumor biology. Indeed, these analyses have demonstrated the occurrence of previously unreported genetic mutations at an early stage of carcinogenesis in patients with luminal A subtype breast cancer [23]. Furthermore, in other studies, researchers showed that there were no similar genomic mutation patterns in hypermutated colorectal cancer and rectal cancer [24]. Thus, it is becoming clear that each cancer type and subtype have unique expression patterns and/or genomic backgrounds, suggesting that known oncogenes and tumor-suppressor genes are crucial for tumor development. Additionally, bioinformatics analysis is becoming increasingly important because it has enabled us to identify oncogenes and cancer therapeutic targets, which may be difficult to identify using conventional molecular biological methods [23,24]. Accordingly, p53 functional experimental screening combined with bioinformatics technology may be useful for rapid identification of p53 regulatory target genes and therapeutic targets via the p53 pathway, which have been difficult to identify using conventional experimental methods or bioinformatics analysis alone.

In this study, we conducted p53-associated cell death screening combined with bioinformatics analysis to determine gene expression profiles in patients with cancer using the GEO. After sequential screening, we identified candidate 40 prognostic factors associated with p53 function in human cancer.

## RESULTS

### Combination of *in vitro* and *in silico* analyses for novel genes related to the p53 pathway

**Figure 1:**
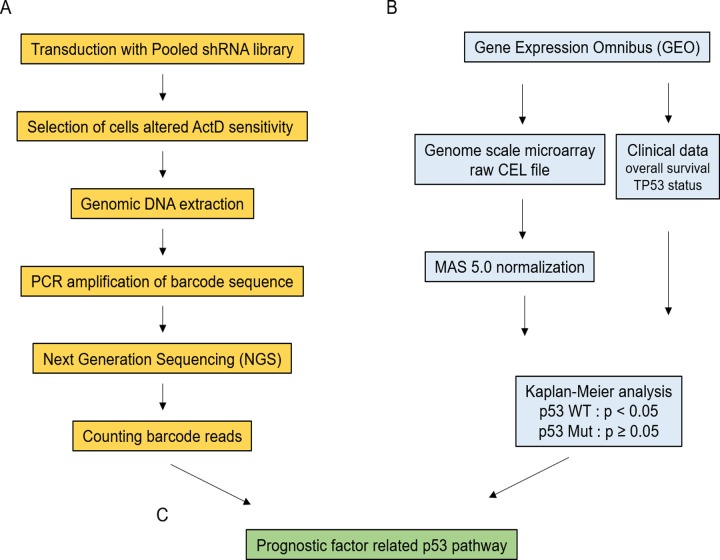
Flowchart of prognosis-related gene identification for targets involved in the p53 pathway by combining pooled shRNA library screening and bioinformatics analysis **(A)** Pooled shRNA library screening for identification of ActD- sensitive or -resistant genes. *TP53* wild-type cancer cells were transduced with a pooled shRNA library. Cells showing increased or decreased ActD sensitivity by gene silencing were selected following ActD stimulation. Genomic DNA from selected cells was extracted. The shRNA barcode sequence inserted into genomic DNA was amplified by PCR. Amplified barcode reads were counted using NGS. The same procedures were carried out for DMSO-treated cells as controls. The fold-change (FC) of barcode reads was calculated. **(B)** Survival analysis of patients with cancer based on *TP53* mutational status. The datasets for the human colorectal cancer DNA microarray (GSE39084 and GSE39582) were obtained from the GEO. Each raw CEL file was normalized to MAS 5.0. Probes used to measure gene expression were grouped based on the median level of gene expression in *TP53* wild-type (WT) and mutant (Mut) cancers. The overall survival of patients with cancer was estimated by the Kaplan-Meier method. Significant differences were analyzed using log-rank tests (p < 0.05). Genes with significant difference in *TP53* WT samples but without significant difference in *TP53* Mut samples were selected. **(C)** Genes that showed altered ActD sensitivity in pooled shRNA library screening (A) were combined with genes that affected prognosis in a p53-dependent manner (B). Genes included in both lists were selected as prognostic factors related to the p53 pathway.

First, we performed RNAi screening using a lentiviral shRNA library to identify genes related to p53- dependent apoptosis (Figure 1A). This library comprised 27,500 shRNAs targeting approximately 5,000 genes. Each shRNA had a unique barcode sequence that could be used to identify the inserted shRNA. When infecting cells with lentivirus at a multiplicity of infection (MOI) of 0.1, the possibility that two or more lentiviral particles infected a single cell was less than 5%; thus, lentiviral dual infection events were suppressed [25]. Based on this result, p53 wild-type U2OS human osteosarcoma cells were infected with lentivirus carrying shRNA at an MOI of 0.1 in order to avoid multiple lentiviral infections.

The genes selected by shRNA library screening included genes controlling cell death and cell cycle, regardless of the p53 pathway. Therefore, prognostic factors in colorectal cancer depending on p53 mutational status were selected from the screened genes because only wild-type p53 regulated prognostic factors related to alteration of overall survival (Figure 1B).

We expect to identify prognostic genes related to the p53 pathway via a combination of pooled shRNA library screening to select genes that are sensitive or resistant to p53-activating drugs and bioinformatics analysis to assess the effects of genes on the survival of patients with cancer depending on p53 mutational status (Figure 1C).

### Pooled shRNA library screening

**Figure 2:**
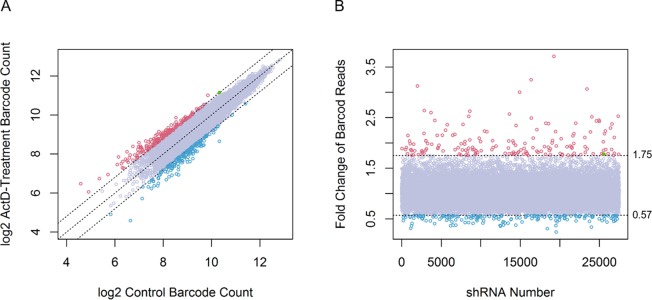
Selection of candidate p53-regulatory genes or p53 target genes by pooled shRNA library screening **(A)** *TP53* wild-type U2OS cells were transduced with pooled shRNA library by lentivirus infection. After puromycin selection, infected cells were treated with ActD or DMSO as a control for 3 days. Genomic DNA was extracted from ActD-treated U2OS cells. The barcode sequence of the inserted shRNA was analyzed by NGS to count the barcode reads. Barcode read counts were log2 transformed. The plot indicates correlations of barcode reads in ActD- or DMSO-treated samples. Red circles, shRNAs targeting ActD-sensitive genes; blue circles, shRNAs targeting ActD-resistant genes; filled green circle, shRNAs targeting *TP53*. **(B)** Fold-changes in barcode reads for each shRNA. Red circles indicate 1.75-fold changes in shRNA barcode reads. Blue circles indicate less than 0.57-fold changes in shRNA barcode reads. Filled green circle indicates *TP53* shRNA.

In order to detect shRNA enrichment or depletion, shRNA barcodes from ActD-stimulated cells and DMSO- treated cells as controls were amplified and counted by NGS. Barcode reads in the ActD-stimulated and control groups were normalized such that the barcode read ratios of both groups could be accurately estimated (Figure 2A). One-hundred sixty-three shRNAs targeting 161 genes showed 1.75-fold change in barcode counts (Figure 2B). We designated these genes as ActD-sensitive genes. Additionally, 164 shRNAs targeting 161 genes showed less than 1/1.75 (0.57)-fold change in barcode counts (Figure 2B). We designated these genes as ActD-resistant genes (Figure 2B).

**Figure 3:**
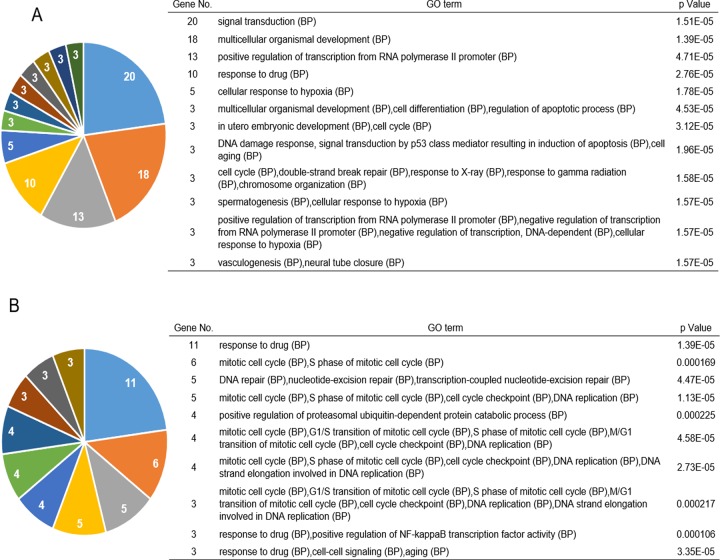
Functional analysis of genes identified in the shRNA library screening. Results of Gene Ontology (GO) enrichment analysis. **(A)** ActD-sensitive and **(B)** ActD-resistant genes identified by shRNA screening were analyzed using GeneCodis3 to identify the related biological processes. Significantly enriched genes are shown. (A) *p* ≤ 4.70713 × 10^−5^, (B) p ≤ 2.24936 × 10^−4^.

We conducted functional enrichment analysis with GeneCodis 3 (http://genecodis.cnb.csic.es) to evaluate the biological processes of ActD-sensitive and ActD-resistant genes. ActD-sensitive genes were involved in biological processes such as response to drug, DNA damage response, and cell cycle (Figure 3A). ActD-resistant genes were involved in biological functions such as drug response, mitotic cell cycle, and DNA repair (Figure 3B). Notably, p53 shRNA was screened as targeting an ActD- sensitive gene (Figure 2B).

Because the screened genes were involved in cell protection, similar to p53 function, and p53 shRNA was selected, the screening selected genes related to the p53 pathway.

### Analysis of microarray data

In our shRNA library screening, we assumed that ActD-sensitive and -resistant genes included genes related to the p53 pathway as well as genes that were not related to the p53 pathway but were still involved in cell viability. To select potential target genes related to the p53 pathway based on ActD sensitivity and resistance, we estimated the duration of survival for patients with colorectal cancer with or without *TP53* mutation using GEO datasets.

**Figure 4:**
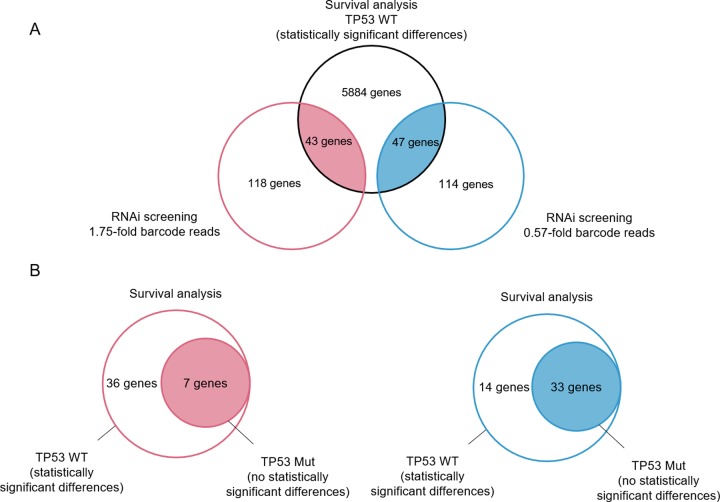
Identification of genes associated with survival in patients with cancer with regard to p53 mutation status using bioinformatics. **(A)** Venn diagram indicating the overlap of genes related to survival in patients with *TP53* WT colorectal cancer and genes selected by shRNA library screening. Patient survival was estimated using the Kaplan-Meier method. Statistical significance was determined by log-rank tests (*p* < 0.05). Red, 1.75-fold changes in barcode reads indicating ActD-sensitive genes; blue, less than 0.57- fold changes in barcode reads indicating ActD-resistant genes. **(B)** Venn diagram indicating overlap of genes not related to prognoses in colorectal cancer specimens with *TP53* Mut and genes in (A). Red, ActD-sensitive genes; blue, ActD-resistant genes. Survival analysis and statistical tests were the same as in (A).

Microarray raw CEL files of 421 patients with colorectal cancer originated from GEO datasets (*TP53* wild-type: 200; *TP53* mutant: 221). After MAS5.0 normalization and quality control, 418 datasets were obtained (*TP53* wild-type: 197; *TP53* mutant: 221). We estimated colorectal cancer prognoses for each gene using Kaplan-Meier analysis with R to assess patient survival visually and statistically. Patients with colorectal cancer were divided into high and low expression groups for every gene based on the median signal intensity of each probe. As a result, 5974 genes (9258 probes) were found to be related to survival in TP53 wild-type colorectal cancer (Figure 4A).

Apoptosis-related genes involved in the p53 pathway selected by RNAi screening were expected to induce or suppress cell death following ActD stimulation. Similar to p53, high expression of ActD-sensitive genes was found to improve prognosis in patients with *TP53* wild-type cancer. Conversely, survival was worse in the context of high expression of ActD-resistant genes in patients with *TP53* wild-type cancer. Thus, we chose genes that were significantly correlated with prognosis from ActD-sensitive or -resistant genes in patients harboring wild-type *TP53*. In these patients, 43 ActD-sensitive genes and 47 ActD-resistant genes significantly altered prognosis (Figure 4A). We assumed that these genes did not alter survival outcomes significantly in patients with *TP53* mutant cancer because mutant *TP53* enhanced malignant progression. Therefore, we selected genes that did not alter survival in patients with mutant *TP53*. From this analysis, 7 ActD-sensitive genes and 33 ActD-resistant genes showed a significant prognostic correlation in patients with *TP53* wild-type colorectal cancer, but no significant prognostic correlation in patients with the *TP53* mutant (Figure 4B).

**Figure 5:**
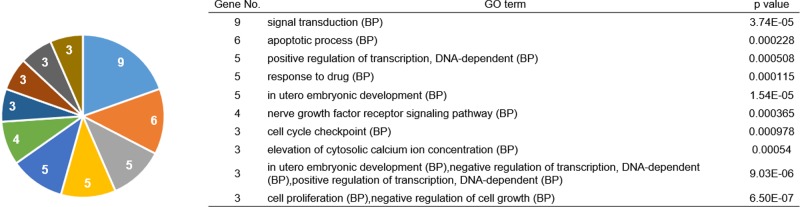
Functional analysis of genes selected by pooled shRNA library screening and survival analysis Results of Gene Ontology (GO) enrichment analysis for 40 genes related to the survival of patients with *TP53* wild-type colorectal cancer and genes not related to prognosis in patients with *TP53* mutant cancer selected by shRNA library screening. Enrichment analysis of biological processes was performed using GeneCodis 3. Significantly enriched genes are shown (*p* ≤ 0.00097773).

Finally, 40 genes were analyzed for function using Gene Ontology analysis. These genes were found to be associated with p53-related functions, including apoptotic process, drug response, cell cycle checkpoint, and cell proliferation (Figure 5).

## DISCUSSION

In the present study, we selected 40 genes that altered survival prognosis in patients with colorectal cancer in a p53 function-dependent manner by combining shRNA library screening and database analysis of gene expression. Many genes selected by this combination method were involved in processes relevant to the p53 pathway, including apoptosis, drug response, cell cycle checkpoint, and cell proliferation. In fact, these genes included known p53 pathway genes, such as *CCNB1* [26], *ANKHD1* [27], nuclear receptor coactivator 3 (*NCOA3*) [28], *SIPA1* [29], *IKBKB* [30], and *LITAF* [31]. Consequently, this method combining *in vitro* shRNA library screening with *in silico* survival prognostic analysis was found to be effective for selection of genes related to the p53 pathway. We also examined whether this method could be used to identify prognostic factors related to the p53 pathway in patients with cancer. Importantly, *TP53* status has been shown to affect prognosis in patients with colon cancer because patients with p53 mutant colorectal cancer have poorer survival outcomes than patients with p53 wild-type cancer [32,33]. In addition, the gene expression level of the p53 pathway regulator *PICT1* has been correlated with prognosis in patients with p53 wild- type cancer [18-21]. Thus, a method based on analysis of p53 mutational status-dependent survival in patients with cancer may be useful for effective identification of survival factors via the p53 pathway.

**Figure 6:**
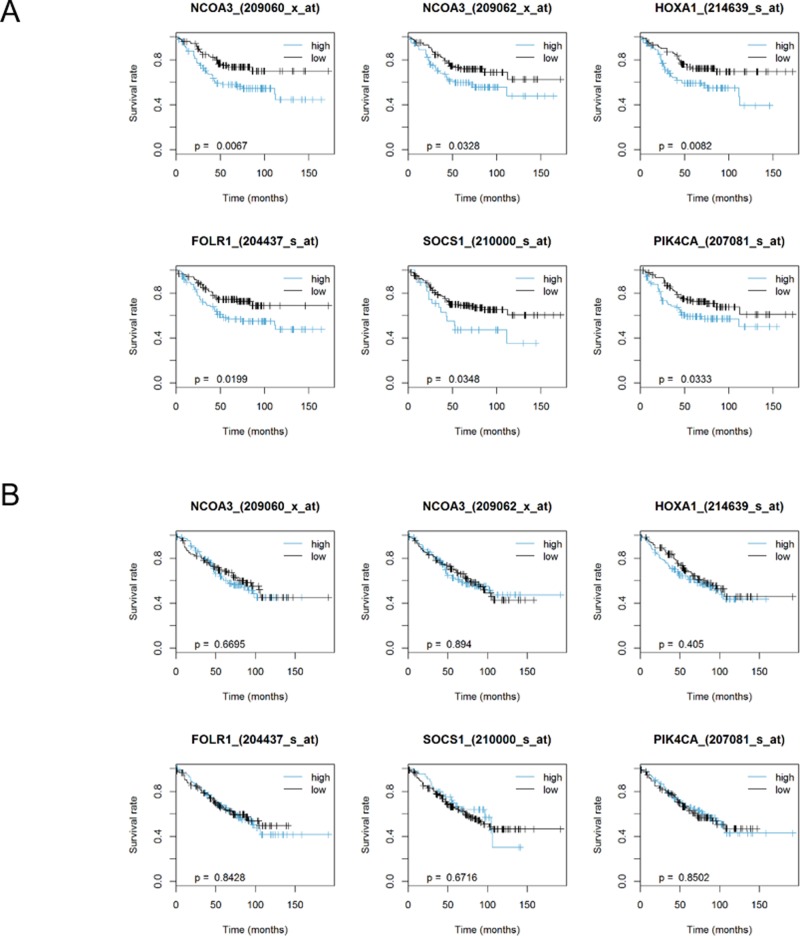
Expression of selected candidate genes influenced the survival of patients with colorectal cancer in a p53- dependent manner Kaplan-Meier survival plots for patients with (A) *TP53* WT and (B) Mut colorectal cancer to determine the effects of selected genes (*NCOA3*, *HOXA1*, *FOLR1*, *SOCS1*, and *PIK4CA*) selected by shRNA library screening and database analysis on survival. Log rank tests were used for statistical analysis (*p* < 0.05).

Of the ActD-sensitive genes selected by shRNA library screening, high expression of seven genes showed a better prognosis in patients with p53 wild-type colorectal cancer. Moreover, high expression of 33 ActD-resistant genes was associated with poor prognosis in patients with *TP53* wild-type cancer (Figure 6A, Supplementary Figure 1). All 40 genes, however, did not affect prognosis in patients with *TP53* mutant cancer (Figure 6B, Supplementary Figure 1), demonstrating a dependence on *TP53* mutational status. The genes selected using this method included *NCOA3* [34,35], *HOXA1* [36], *FOLR1* [37], *SOCS1* [38], and *PIK4CA* [39], which showed similar expression patterns related to survival in patients with cancer. This result suggested that our method was a relatively simple method for identification of genes functionally associated with the p53 pathway.

Notably, the *NCOA3* gene identified by our combination method was shown to be involved in both regulation of the p53 pathway and determination of survival in patients with cancer. NCOA3 protein promotes the transcription of the *TRAF4* gene, which encodes tumor necrosis factor receptor-associated factor 4 (TRAF4). High TRAF4 expression induces downregulation of p53 because TRAF4 destabilizes p53 by inhibiting the p53 deubiquitinase herpesvirus-associated ubiquitin-specific protease [28]. Furthermore, patients with breast cancer with high *NCOA3* expression have a poorer prognosis than patients with low *NCOA3* expression [34,35]. In our *in silico* analysis of a colorectal cancer gene expression database, the expression detected by two independent microarray probes for *NCOA3* indicated a significant correlation with survival in patients with colorectal cancer depending on *TP53* genotype status; thus, these results suggest that *NCOA3* was a prognostic factor via the p53 pathway. These findings strongly support that our method was an excellent approach for selecting prognostic factors regulated by the p53 pathway in patients with cancer (Figure 6A, 6B, Supplementary Figure 1).

In this study, we provided a convenient method to identify prognostic factors functionally associated with p53 from a large number of candidate genes. In many studies, several genes involved in the p53 pathway have been identified *in vitro* [40], *in vivo* [41], or both. By conventional methods, genes of interest have been used to investigate phenotypic changes in cells and model organisms, such as knockout mice. However, these methods are expensive and time consuming owing to the processes of genetic engineering and construction of analytical systems. Accordingly, it has been difficult to identify genes involved in the p53 pathway from many candidate genes within a short period of time. Moreover, functional analyses of individual genes are still required in order to identify prognostic factors associated with the tumor-suppressor function of p53. These analyses are also limited by the high cost and time-consuming procedures and are therefore not convenient to perform. Furthermore, false-positive genes in candidates selected through large- scale functional screening with library expression analysis can make it difficult to identify true-positives.

In this study, we provided a simple and highly accurate technology to estimate candidate gene functionally related to the p53 pathway without interference of false-positive genes and without the need to perform validation of individual genes. We used osteosarcoma U2OS cells *in vitro* and patients with colorectal cancer *in silico* in our screening because U2OS cells are suitable for the study of p53 signaling and there is sufficient data for a number of patients associated with p53 mutational status in colorectal cancer available in the GEO database for technical issues. We believe our method using two different types of cancer is suitable for isolation of p53-signaling genes for the following two reasons: 1) to a considerable extent, p53 signaling is common between osteosarcoma and colorectal cancer [40,42]; and 2) using different types of cancer may generally reduce the screening biases generated by a specific single cancer cell type. Furthermore, our method may be applicable to other genes, such as *HER2* amplification in patients with breast cancer, because patients with breast cancer and amplified *HER2* have poor survival compared to those patients with normal *HER2* [43]. However, some genes selected in this study have not been reported to be involved in p53 regulation or survival in patients with cancer. Thus, further functional validation of these genes is needed. Identification of prognostic factors via the p53 pathway may lead to the identification of novel therapeutic target genes in patients with cancer.

In conclusion, we established a combination method with large-scale shRNA library screening *in vitro* and *in silico* analysis of gene expression databases for patients with cancer to identify prognostic factors based on a functional biological pathway. Our method established a new technology for the identification of prognostic factors associated with the p53 pathway in patients with cancer, enabling analysis of thousands of genes at one time. The genes selected by this method affected the survival of patients with cancer. Thus, these genes may be novel targets of anticancer drugs.

## MATERIALS AND METHODS

### Cell culture and treatments

U2OS and HEK293FT cells were obtained from the American Type Culture Collection (Manassas, VA, USA) and Thermo Fisher Scientific (Waltham, MA, USA), respectively. Cells were cultured in Dulbecco's modified Eagle's medium (DMEM; Nissui, Tokyo, Japan) containing 10% fetal bovine serum in an atmosphere containing 5% CO2 at 37°C with 100% humidity.

### Preparation of lentivirus

HEK293FT cells (6.0 × 106 cells) were cultured in 10-cm dishes and cultured for 1 day. The plasmids pMDLg/pRRE (6.5 µg), pRSV-Rev (2.5 µg), pMD2.G (3.5 µg), and 2.5 µg of Human DECIPHER Module 1 (Cellecta, Mountain View, CA, USA), i.e., lentivirus vectors expressing 27,500 shRNAs targeting approximately 5,000 genes involved in cell signaling, were mixed in 500 µL serum-free DMEM. Separately, 20 µL of Lipofectamine 2000 (Thermo Fisher Scientific) was mixed with 500 µL serum-free DMEM. These two solutions were mixed. Then, HEK293FT cells were transfected with the plasmid mixture and cultured for 2.5 days. The cultured medium was centrifuged at 3,500 rpm for 5 min, and the supernatant containing lentiviral particles was then harvested. The supernatants were stored at -80°C.

Because Human DECIPHER Module 1 contained the red fluorescent protein (RFP) coding sequence, cells infected with lentivirus expressed RFP. To determine the lentiviral titer, the numbers of U2OS cells expressing RFP and of 4',6-diamidino-2-phenylindole dihydrochloride (DAPI; Dojindo, Kumamoto, Japan)-positive U2OS cells were counted after lentiviral infection. U2OS cells were plated in 6-well plates at 5.0 × 105 cells/well. The prepared lentivirus stock solution was serially diluted twice in DMEM to a final volume of 500 µL. The solutions were then added to the cells. After 2 days, lentivirus-infected cells were stained with DAPI diluted 4,000 times for 10 min. The cells were observed with a confocal laser scanning microscope (LSM 700; Carl Zeiss, Oberkochen, Germany). Cells stained with DAPI and expressing RFP were counted as the total number of cells and the number of cells harboring shRNA, respectively. A dilution ratio close to 10% was defined as an MOI of 0.1.

### Genome-wide RNAi screening

For RNAi screening, 3.0 × 106 U2OS cells were plated in 8.9 mL DMEM in 20 dishes (10-cm) and infected with lentiviral particles at an MOI of 0.1. Since lentivirus-infected cells exhibited resistance to puromycin (Invivogen, San Diego, CA, USA), the cells were selected with 2.5 µg/mL puromycin. After 2 days, puromycin-selected cells were stimulated with 7.5 nM ActD (Wako, Osaka, Japan; the half-maximal inhibitory concentration [IC50]) for 3 days in order to induce p53 hyperactivation-related apoptosis. Genomic DNA was isolated from stimulated cells, and the barcode sequence was amplified by PCR. Amplified fragments were sequenced by NGS (Cosmo Bio, Tokyo, Japan), and changes in ActD sensitivity were evaluated by counting shRNA barcode reads. Target gene silencing by shRNA was found to promote apoptosis through p53 when the number of barcode reads in the ActD-stimulated group was greater than that in the control group. Conversely, target gene silencing by shRNA was found to suppress apoptosis through p53 when the number of barcode reads in the ActD-stimulated group was lesser than that in the control group.

### Collection and analysis of human colon and colorectal cancer microarray data

The datasets from GSE 39084 and GSE 39582 (*TP53* wild-type: 200 specimens, *TP53* mutant: 221 specimens, *TP53* mutation unknown: 215 specimen) were downloaded from GEO. The raw CEL files were normalized by MAS 5.0 using an Expression Console (Affymetrix, Thermo Fisher Scientific) with an Affymetrix mask file. Quality control (background < 120, RawQ < 10, percent present calls < 55, scaling factor < 3, GAPDH 3′ to 5′ ratio < 5, beta-actin 3′ to 5′ ratio < 5) was performed. Probe signal intensities were converted to log2 values with R. After log2 conversion, probes with zero values were excluded.

### Survival analysis

Patients with *TP53* wild-type or mutant colorectal cancer were divided into high and low expression groups according to the median signal intensity of each microarray probe. The overall survival in the two groups was compared by the Kaplan-Meier method using the R-3.1.2 and “survival” packages. Significant differences in survival were calculated by log-rank tests and defined as having a *P* value of less than 0.05. This analysis was performed in patients with *TP53* wild-type and mutant cancer.

### Functional analysis of prognostic factors

Gene Ontology analysis was performed using GeneCodis 3 (http://genecodis.cnb.csic.es/analysis). Annotations were conducted with GO biological processes. *HILPDA* (also called *HIG2*), *RPS17P16* (also called *LOC402057*), and *CD31* (also called *PECAM1*) were not registered in GeneCodis 3 and were therefore excluded from this analysis.

## SUPPLEMENTARY MATERIALS


