# Supplementary material for: A novel isolation method for cancer prognostic factors via the p53 pathway by a combination of in vitro and in silico analyses
Source: Oncoscience. 2018 Apr 29;5(3-4):88–98. doi: 10.18632/oncoscience.411 (PMC5978436; doi:10.18632/oncoscience.411)
Supplement: Supplementary file 1 [file oncoscience-05-0088-s001.pdf]

SUPPLEMENTARY MATERIALS

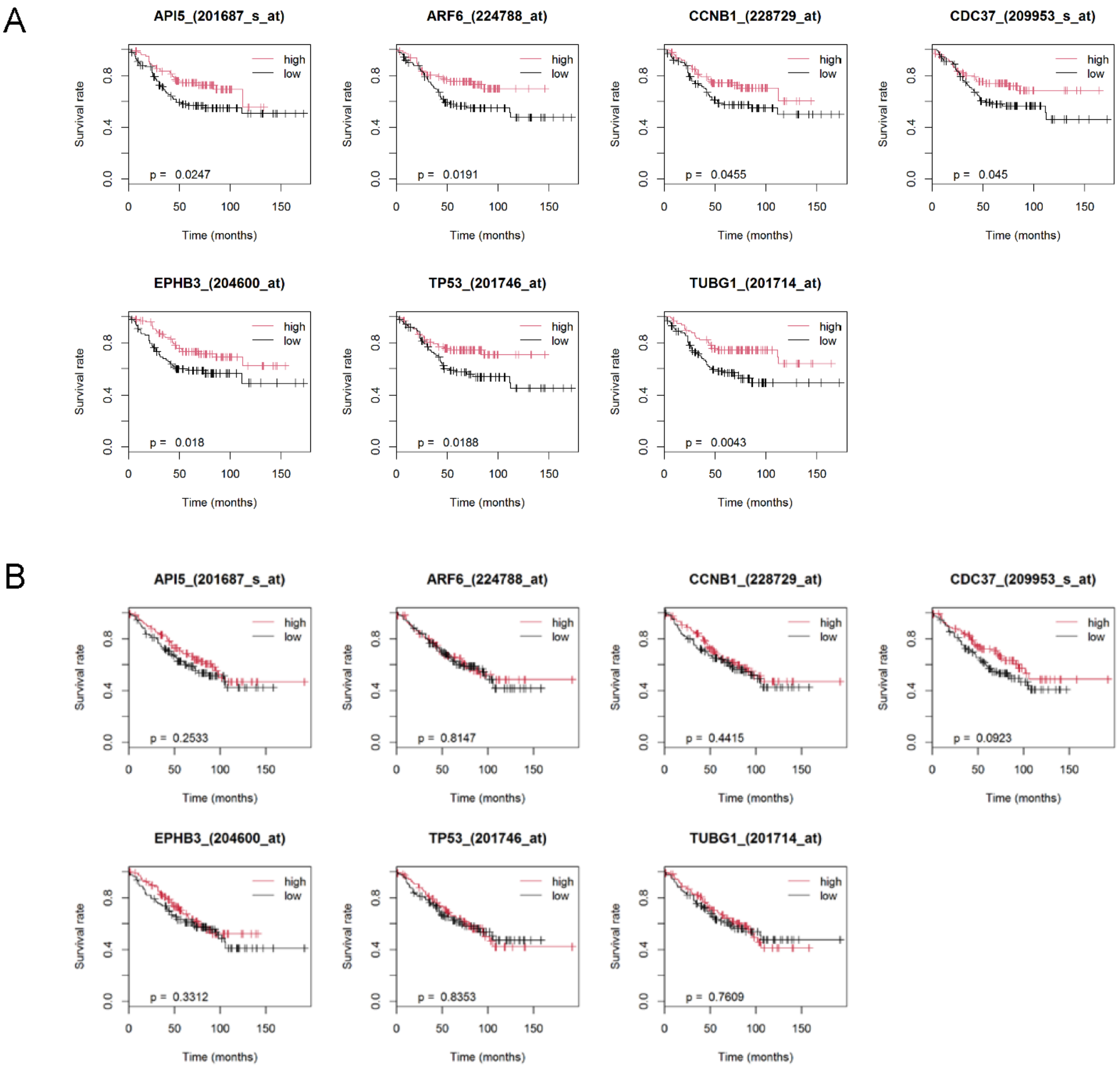

(Continued)

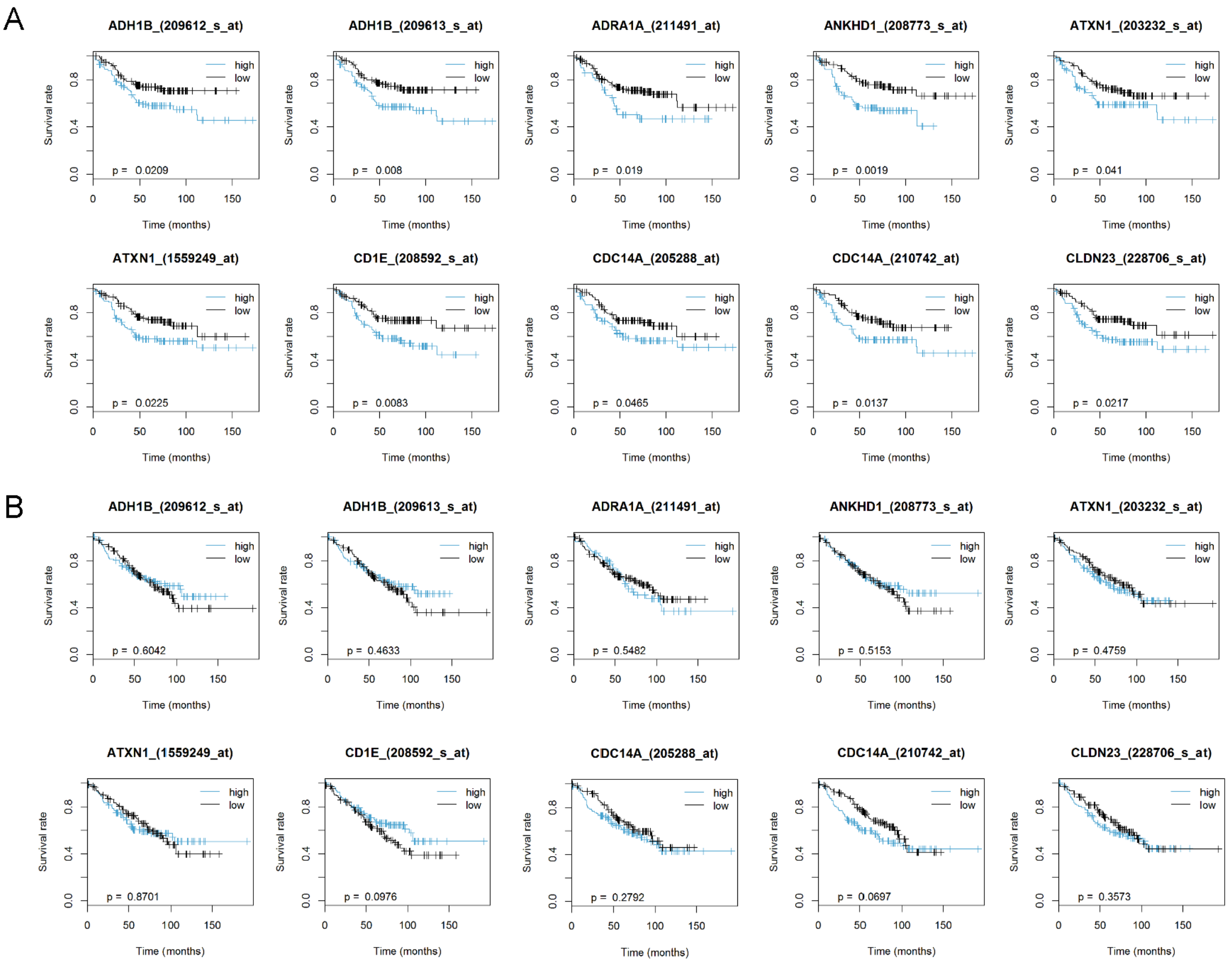
A
ADH1B_(209612_s_at)
p = 0.0209
ADH1B_(209613_s_at)
p = 0.008
ADRA1A_(211491_at)
p = 0.019
ANKHD1_(208773_s_at)
p = 0.0019
ATXN1_(203232_s_at)
p = 0.041
ATXN1_(1559249_at)
p = 0.0225
CD1E_(208592_s_at)
p = 0.0083
CDC14A_(205288_at)
p = 0.0465
CDC14A_(210742_at)
p = 0.0137
CLDN23_(228706_s_at)
p = 0.0217
B
ADH1B_(209612_s_at)
p = 0.6042
ADH1B_(209613_s_at)
p = 0.4633
ADRA1A_(211491_at)
p = 0.5482
ANKHD1_(208773_s_at)
p = 0.5153
ATXN1_(203232_s_at)
p = 0.4759
ATXN1_(1559249_at)
p = 0.8701
CD1E_(208592_s_at)
p = 0.0976
CDC14A_(205288_at)
p = 0.2792
CDC14A_(210742_at)
p = 0.0697
CLDN23_(228706_s_at)
p = 0.3573
Survival rate
Time (months)
high
low

(Continued)

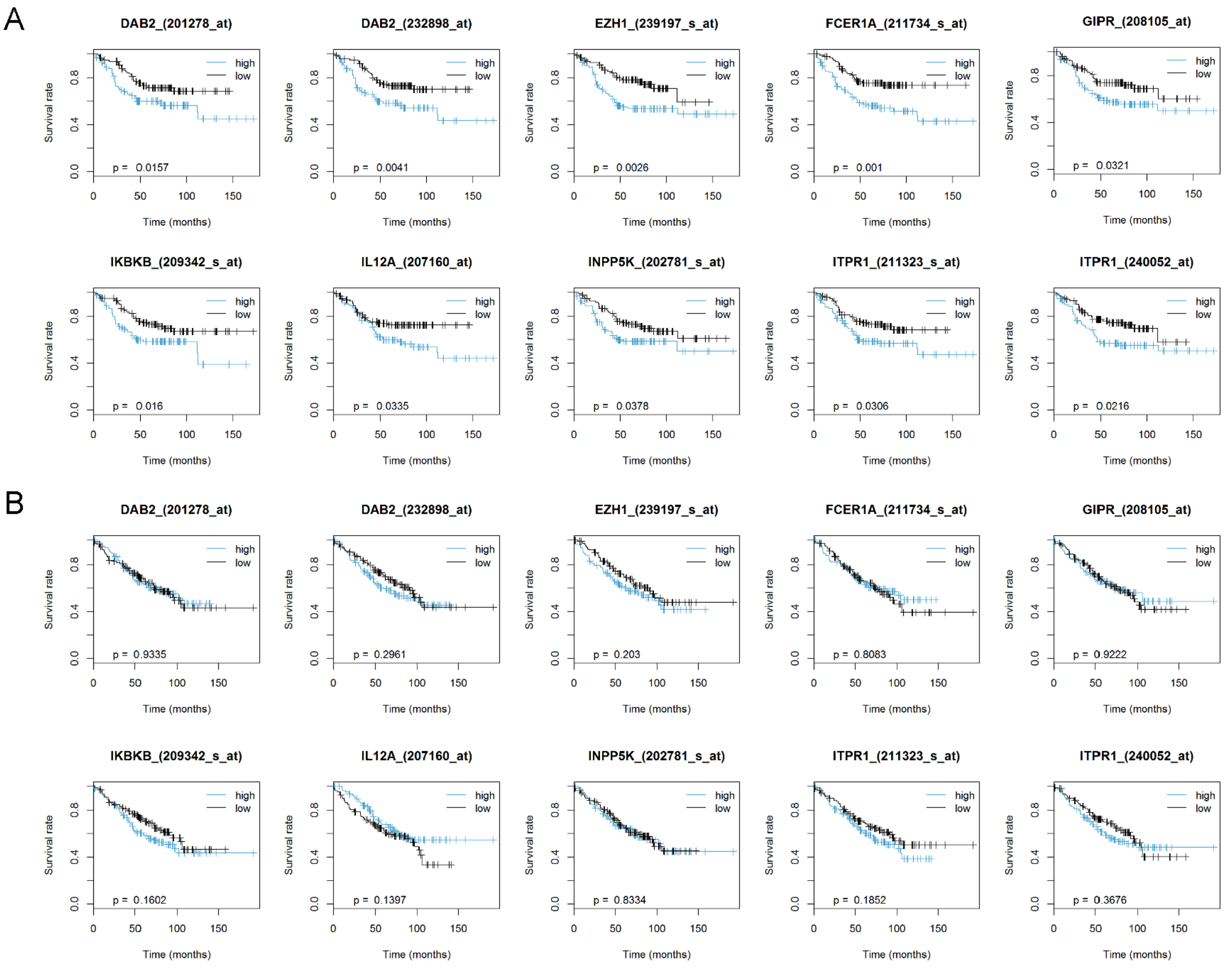
A
DAB2_(201278_at)
p = 0.0157
DAB2_(232898_at)
p = 0.0041
EZH1_(239197_s_at)
p = 0.0026
FCER1A_(211734_s_at)
p = 0.001
GIPR_(208105_at)
p = 0.0321
IKBKB_(209342_s_at)
p = 0.016
IL12A_(207160_at)
p = 0.0335
INPP5K_(202781_s_at)
p = 0.0378
ITPR1_(211323_s_at)
p = 0.0306
ITPR1_(240052_at)
p = 0.0216
B
DAB2_(201278_at)
p = 0.9335
DAB2_(232898_at)
p = 0.2961
EZH1_(239197_s_at)
p = 0.203
FCER1A_(211734_s_at)
p = 0.8083
GIPR_(208105_at)
p = 0.9222
IKBKB_(209342_s_at)
p = 0.1602
IL12A_(207160_at)
p = 0.1397
INPP5K_(202781_s_at)
p = 0.8334
ITPR1_(211323_s_at)
p = 0.1852
ITPR1_(240052_at)
p = 0.3676
high
low
Survival rate
Time (months)

(Continued)

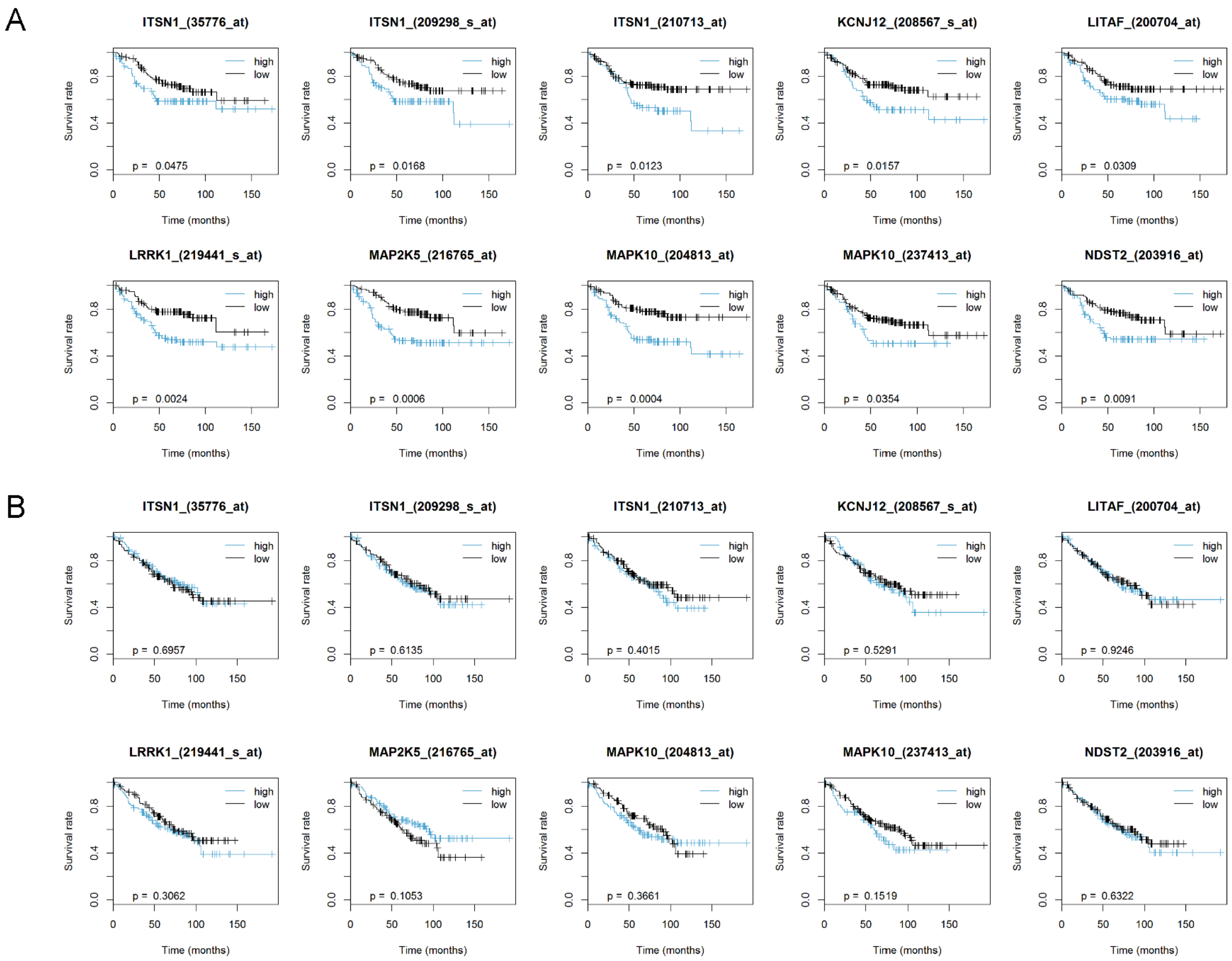
A
ITSN1_(35776_at)
p = 0.0475
ITSN1_(209298_s_at)
p = 0.0168
ITSN1_(210713_at)
p = 0.0123
KCNJ12_(208567_s_at)
p = 0.0157
LITAF_(200704_at)
p = 0.0309
LRRK1_(219441_s_at)
p = 0.0024
MAP2K5_(216765_at)
p = 0.0006
MAPK10_(204813_at)
p = 0.0004
MAPK10_(237413_at)
p = 0.0354
NDST2_(203916_at)
p = 0.0091
B
ITSN1_(35776_at)
p = 0.6957
ITSN1_(209298_s_at)
p = 0.6135
ITSN1_(210713_at)
p = 0.4015
KCNJ12_(208567_s_at)
p = 0.5291
LITAF_(200704_at)
p = 0.9246
LRRK1_(219441_s_at)
p = 0.3062
MAP2K5_(216765_at)
p = 0.1053
MAPK10_(204813_at)
p = 0.3661
MAPK10_(237413_at)
p = 0.1519
NDST2_(203916_at)
p = 0.6322
high
low
Survival rate
Time (months)

(Continued)

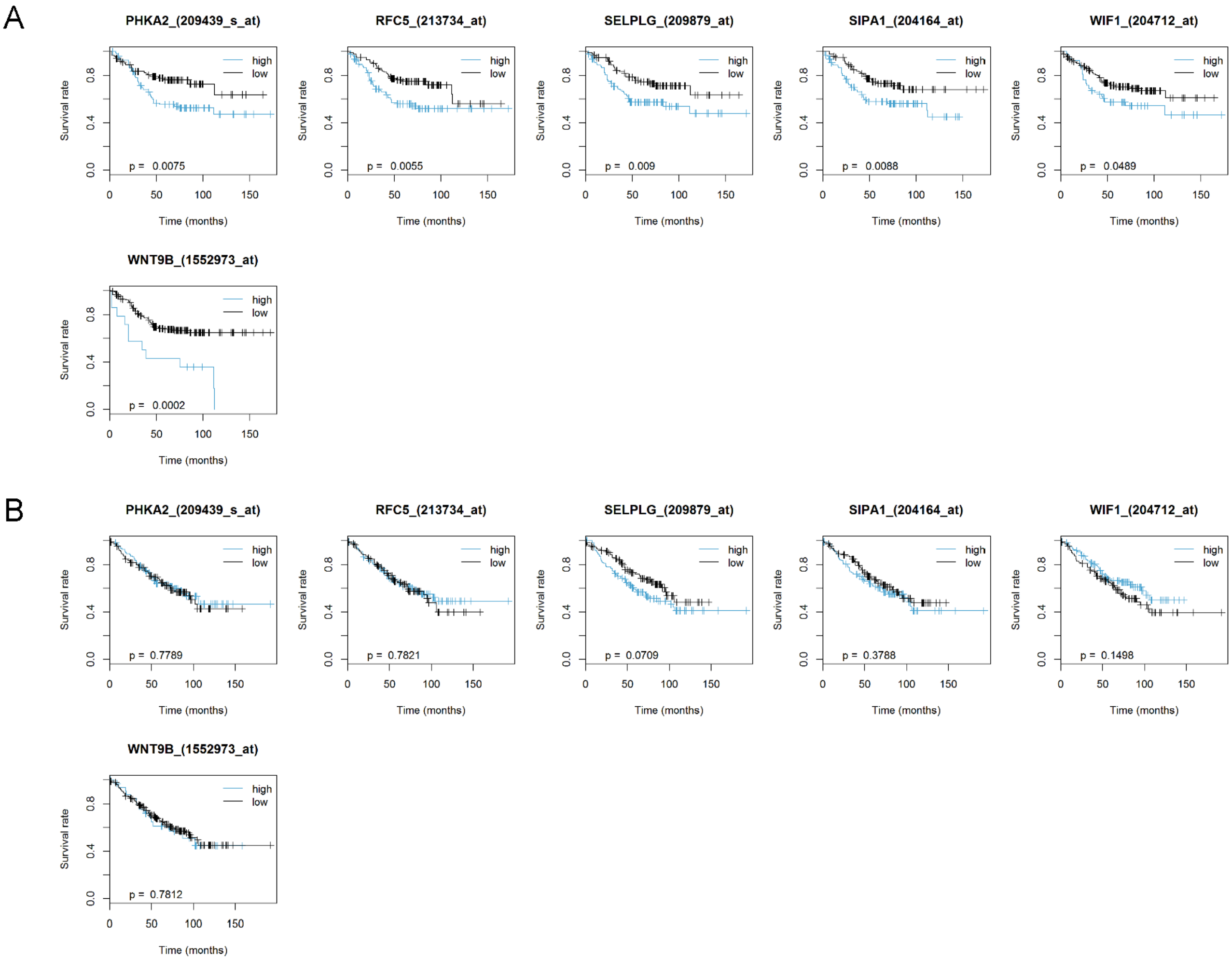

Supplementary Figure 1: Supplementary figure related to Figure 6. Kaplan-Meier survival plots for patients with **(A)** *TP53* WT and **(B)** Mut colorectal cancer to determine the effects of selected genes other than *NCOA3*, *HOXA1*, *FOLR1*, *SOCS1*, and *PIK4CA* selected by shRNA library screening and database analysis on survival. Log rank tests were used for statistical analysis ($p < 0.05$). Red and black line, ActD-sensitive genes; blue and black line, ActD-resistant genes.
